# Caregiver burden and health-related quality of life: A study of  informal caregivers of older adults in Ghana

**DOI:** 10.1186/s41043-024-00509-3

**Published:** 2024-02-21

**Authors:** Williams Agyemang-Duah, Alhassan Abdullah, Mark W. Rosenberg

**Affiliations:** 1 Department of Geography and Planning, Queen’s University, Kingston, ON K7L 3N6 Canada; 2 College of Education, Psychology and Social Work, Flinders University, Adelaide, SA Australia

**Keywords:** Caregiver burden, Health-related quality of life, Informal caregivers, Older adults, Ghana

## Abstract

**Background:**

Similar to many developing countries, caregiver burden remains high in Ghana which may affect informal caregivers of older adults’ health-related quality of life (HRQoL). However, no study has examined the association between caregiver burden and HRQoL among informal caregivers of older adults in Ghana to date. Understanding this association may well help to inform health and social policy measures to improve HRQoL among  informal caregivers of older adults in Ghana. Situated within a conceptual model of HRQoL, the purpose of this study was to examine the relationship  between caregiver burden and HRQoL among informal caregivers of older adults in Ghana.

**Methods:**

We obtained cross-sectional data from informal caregiving, health, and healthcare (*N* = 1853) survey conducted between July and September 2022 among caregivers (≥ 18 years) of older adults (≥ 50 years) in the Ashanti Region of Ghana. The World Health Organization Impact of Caregiving Scale was used to measure caregiver burden. An 8-item short form Health Survey scale developed by the RAND Corporation and the Medical Outcomes Study was used to measure HRQoL. Generalized Linear Models were employed to estimate the association between caregiver burden and HRQoL. Beta values and standard errors were reported with a significance level of  0.05 or less.

**Results:**

The mean age of the informal caregivers was 39.15 years and that of the care recipients was 75.08 years. In our final model, the results showed that caregiver burden was negatively associated with HRQoL (*β* = − .286, SE = .0123, *p* value = 0.001). In line with the conceptual model of HRQoL, we also found that socio-economic, cultural, demographic and healthcare factors were significantly associated with HRQoL. For instance, participants with no formal education (*β* = −1.204, SE= .4085, *p* value = 0.01), those with primary level of education (*β* = −2.390, SE= .5099, *p* value = 0.001) or junior high school education (*β* = −1.113, SE= .3903, *p* value= 0.01) had a significantly decreased HRQoL compared to those with tertiary level of education. Participants who were between the ages of 18–24 (*β* = 2.960, SE= .6306, p value=0.001), 25–34 (*β* = 1.728, SE= .5794, *p* value = 0.01) or 35–44 (*β* = 1.604, SE= .5764, p value= 0.01) years significantly had increased HRQoL compared to those who were 65 years or above. Also, participants who did not utilize healthcare services in the past year before the survey significantly had increased HRQoL compared to those who utilized healthcare services five or more times in the past year (*β* = 4.786, SE=. 4610, *p* value= 0.001).

**Conclusion:**

Consistent with our hypothesis, this study reported a significant negative association between caregiver burden and HRQoL. Our findings partially  support the conceptual model of HRQoL used in this study. We recommend that health and social policy measures to improve HRQoL among  informal caregivers of older adults should consider caregiver burden as well as other significant socio-economic, cultural, demographic, and healthcare factors.

## Introduction

The population of older adults in sub-Saharan Africa (SSA) continues to increase [1] in line with global ageing. The increasing ageing population has implications for the health and healthcare of older adults in SSA including Ghana [2, 3]. A rise in the older adult population in SSA correlates with the  increase in the prevalence of age-related diseases such as hypertension, diabetes, and dementia [4, 5]. Similar to many developing countries, older adults in Ghana experience chronic diseases [2] such as hypertension, arthritis, diabetes [6–8], eye problem/cataracts, stroke, asthma, chronic kidney disease, chronic lung disease, cancer, and ear problems [7, 8]. The increase in chronic diseases among older adults also correlates with higher demand for healthcare services to deal with their health problems [9, 10]. Yet, evidence shows that Ghanaian older adults continue to experience unmet health (care) needs due to individual and systemic barriers to healthcare use [3, 11]. For instance, research continues to demonstrate that older adults encounter transportation, financial, communication and attitudinal problems in their quest to utilize healthcare [12]. As a result of these healthcare utilization challenges, some older people who require formal healthcare find themselves dependent on informal care. Further, many older adults who need residential care also find themselves dependent on informal care because of the limited (although growing) number of residential care homes in developing countries (including Ghana) [13–16].

Defined mostly as an unpaid care provided by family members, friends, and neighbors to persons who require help to manage various activities of daily living (ADL) such as bathing, dressing, and taking medications [17, 18], informal caregiving  continues to rise in Ghana. As in other SSA contexts, informal caregivers perform domestic, healthcare, economic, social, and spiritual responsibilities in Ghana [19]. Notwithstanding these responsibilities, informal caregivers face challenges such as inadequate funds, lack of ability to work effectively, persistent stress, inadequate time for socialization and emotional trauma [19] which expose them to caregiver burden. In this case, caregiver burden is conceptualized as an "emotional, physical and financial demands and responsibilities of an individual’s illness that are placed on members, friends or other individuals involved with the individual outside the healthcare system" [20, p. 12]. Statistics show that the prevalence of caregiver burden is high in SSA. For example, Addo et al. [21] in a systematic review indicated that 71% of informal caregivers of persons with severe mental illness experience economic burden of caregiving in SSA. In Nigeria, the prevalence of caregiver burden is 96.7% [22]. In Ghana, studies report that 74.6% of informal caregivers of stroke patients experience financial burden, 66.9% experience physical burden, 63.6% face psychological burden and 51.7% report social burden [23]. Another Ghanaian study found that place of residence, provision of financial, health and physical supports to care and receipt of financial, physical and health supports explain caregiver burden [24].

The prevalence of caregiver burden affects health-related quality of life in terms of poor physical and psychological health of informal caregivers [25–27]. In an Ethiopian study, 47.5% of family caregivers of individuals with psychiatric illness report a poor quality of life [28]. Despite the high prevalence of caregiver burden in SSA including Ghana, not much is known regarding the association between caregiver burden and health-related quality of life among informal caregivers of older adults. The existing research has focused on family caregivers with schizophrenic patients [29], informal caregiving for children with lymphoma [30] and caregivers of stroke survivors [31]. For instance, Opoku-Boateng et al. [29] wrote on economic cost and quality of life among family caregivers of schizophrenic patients attending psychiatric hospitals in Ghana. Their study reports that caregivers with higher severity of depression, anxiety and stress experience higher caregiver burden and lower quality of life. Another independent study by Dawson et al. [30] focused on costs, burden and quality of life associated with informal caregiving for children with lymphoma attending a tertiary hospital in Ghana. They found that lymphoma is linked to increased cost and higher burden impacting on quality of life among caregivers. Also, the Boakye et al. [31] research on burden of care and quality of life found a positive relationship between strain experience and functional limitations among caregivers of stroke patients. No study, however, has examined the association between caregiver burden and health-related quality of life among informal caregivers of older adults in Ghana.

Understanding the association between caregiver burden and health-related quality of life may well help to inform health and social policy measures to improve the health-related quality of life among informal caregivers of older adults in SSA and Ghana in particular. Such an understanding may also contribute to strengthening efforts to support  informal caregivers of older adults in Ghana.

### Caregiver burden and health-related quality of life

In a published study across six European countries (France, Germany, Italy, Spain, Sweden, and UK), Valcárcel-Nazco et al. [32] found a negative correlation between informal caregivers’ health-related quality of life and caregiver burden. Also, in a cross-sectional study on caregiver burden and health-related quality of life among primary family caregivers of individuals with schizophrenia in Taiwan, Hsiao et al. [33] found that primary family caregivers who experienced mild to moderate caregiver burden had poor health-related quality of life. In a prospective nationwide cohort study on caregiver burden and health-related quality of life among family caregivers of esophageal cancer patients, Schandl et al. [34] found that high-moderate caregiver burden was linked to a decreased health-related quality of life in Sweden. Aside from caregiver burden, demographic and socio-economic factors such as sex, age and living conditions are associated with health-related quality of life among  informal caregivers of patients with amyotrophic lateral sclerosis [35]. Also, marital status, education, literacy, type of caregiver, social support and perceived stigma are associated with quality of life among family caregivers of individuals with mental illness in Ethiopia [28]. A previous Ghanaian study demonstrated that gender, employment status, relationship to care recipients and marital status of the caregivers are associated with quality of life among caregivers of stroke survivors [31].

In SSA, to the best of our knowledge, only one study carried out in Nigeria has been conducted on the influence of caregiver burden and quality of life among informal caregivers of older adults with chronic diseases [36]. In their study, Faronbi and Olaogun [36] report that caregiver burden was associated with health-related quality of life among caregivers of older adults with chronic illness. As a result of the potential differences in caregiver burden, socio-cultural and economic characteristics as well as the healthcare systems between Ghana and Nigeria, there is the need to set up a separate study in Ghana. Apart from that, the Faronbi and Olaogun study focused on informal caregivers providing care for older adults with chronic diseases [36] and not informal caregivers of older adults in general. It is therefore important to broaden the scope of the literature on the association between caregiver burden and health-related quality of life in SSA context by drawing evidence from informal caregivers of older adults with and without chronic diseases. Findings from this study may contribute partly to the realization of the United Nations' health-related Sustainable Development Goals specifically goal three which seeks to achieve health and wellbeing for all at all ages by 2030. To achieve this goal, investigating the relationship  between caregiver burden and health-related quality of life among informal caregivers of older adults is required.

### Conceptual model of health-related quality of life

Situated within the conceptual model of health-related quality of life developed by Ashing-Giwa [37], the objective of this study was to examine the relationship between caregiver burden and health-related quality of life among informal caregivers of older adults in Ghana. The model provides a comprehensive framework to investigate health disparities and risks factors associated with poor outcomes in health-related quality of life research [37]. The conceptual model of health-related quality of life advances the traditional health-related quality of life framework by capturing culturally and socio-ecologically responsive variables [37]. The model is made up of four broad dimensions associated with health-related quality of life: socio-ecological, cultural, demographic, and healthcare [37]. According to Ashing-Giwa [37], the socio-ecological factors comprise socio-economic status (such as education, income, living situation and employment) and life burden (in this case caregiver burden). The cultural factors consist of ethnicity and spirituality (in this case religion). The demographic factors include age, gender, and place of residence. The health system factors focus on access to healthcare and quality of the relationship with health (care) providers [37]. The model thus offers us the opportunity to select our dependent variable (health-related quality of life), independent variables (caregiver burden) and control variables. We hypothesized that caregiver burden would be negatively associated with health-related quality of life among informal caregivers of older adults in Ghana.

## Methods

### Data and sample

**Fig. 1:**
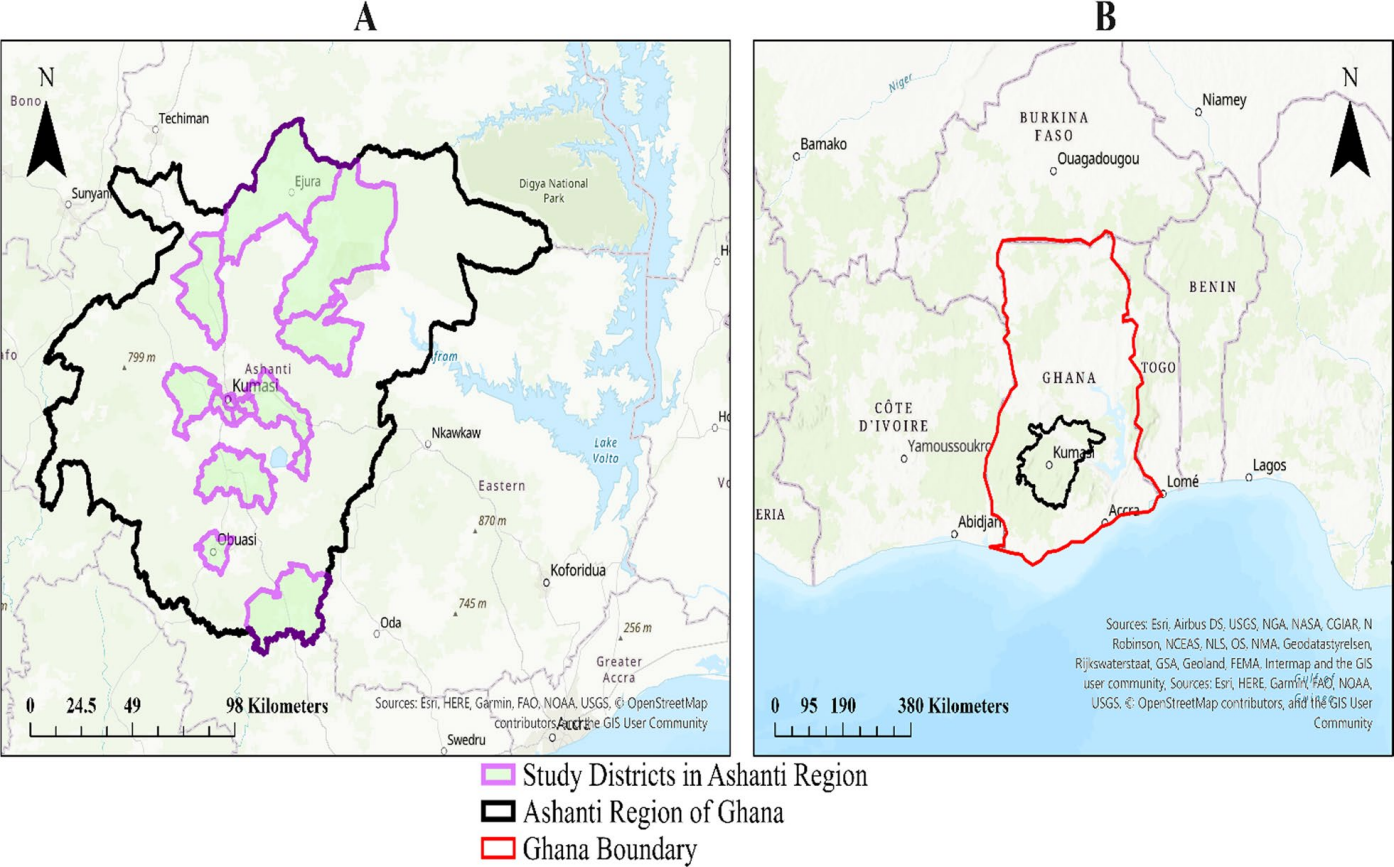
Study area location. **A** Shows the study area covered by the selected districts, and **B** shows the study area in the context of Ghana

Data for this study were derived from a large cross-sectional survey on informal caregiving, health, and healthcare among caregivers (≥ 18 years) of older adults aged 50 years or more. These informal caregivers resided in 13 districts, made up of 18 rural and 21 urban communities, in the Ashanti Region of Ghana (see Fig. 1). Communities and participants in the survey were recruited through probability (simple random and cluster sampling) and non-probability sampling techniques (snowball sampling) respectively. In all, the analytical sample of the survey was 1853 informal caregivers. Details of the sample size calculation and sampling process are found in Agyemang-Duah and Rosenberg [38]. The main data collection instrument was interviewer-administered questionnaire. All the questions captured in the questionnaire were later transferred to Qualtrics, an e-survey tool, to digitally record the responses of the participants. Previous research on the methods of the survey has been reported elsewhere [38].

### Ethics

First, before the fieldwork, we obtained approval to the study site from the Ashanti Regional Health Directorate under the Ghana Health Service (Ref: GHS/ASH/RES/V.2). Second, ethical approval was obtained from the Queen's University General Research Ethics Board (GREB**),** Kingston, Canada (Ref: GGEOPL-344-22**)** and the Committee on Human Research Publication and Ethics (CHRPE), School of Medical Sciences, College of Health Sciences, Kwame Nkrumah University of Science and Technology, Kumasi, Ghana **(**Ref: CHRPE/AP/182/22). Third, both verbal and written informed consents were obtained from the study participants. We declare that the procedure for obtaining the verbal informed consent was approved by the ethics committee/institutional review board. Whereas informed consent for the literate participants was obtained from themselves, that of the illiterate participants was obtained from their legal guardians which was approved by the institutional review board. The ethics protocol emphasized that participation was voluntary and that participants had the right to withdraw from the survey before, during and even after the completion of the survey.

### Dependent variable

In this study, health-related quality of life represents our dependent variable. The Health Survey scale of the health-related quality of life which consists of eight items (SF-8 Health Survey scale), is the shorter version of the 36-item Health Survey scale measuring health-related quality of life [39–43], was employed in this study. The SF-8 Health Survey scale is a general multipurpose short-form health-related quality of life instrument which was developed by the RAND Corporation and the Medical Outcomes Study (MOS) [44]. The SF-8 Health Survey scale has widely been used for health-related quality of life assessment in international health research [39–41, 45–47].

The SF-8 Health Survey scale focuses on 8 domains/dimensions to measure health-related quality of life [48, 49]. These dimensions are physical functioning, physical role functioning, bodily pain, general health, vitality, social functioning, emotional role functioning and mental health [39, 41, 42, 49]. Each domain of the health-related quality of life is linked to a single item on the SF-8 Health Survey scale [40, 41]. These items are as follows: (a) Overall, how would you rate your health during the past 4 weeks? The responses are as follows: (1) very poor, (2) poor, (3) fair, (4) good, (5) very good, and (6) excellent; (b) during the past 4 weeks, how much did physical health problems limit your usual physical activities? The responses are as follows: (1) could not do physical activities, (2) quite a lot, (3) somewhat, (4) very little, and (5) not at all; (c) during the past 4 weeks, how much difficulty did you have in doing your daily work both at home and away from home because of your physical health? The responses are as follows: (1) could not do daily work, (2) quite a lot, (3) somewhat, (4) a little bit, and (5) none at all; (d) how much bodily pain have you had during the past 4 weeks? The responses are as follows: (1) very severe, (2) severe, (3) moderate, (4) mild, (5) very mild, and (6) none; (e) During the past 4 weeks, how much energy did you have? The responses are as follows: (1) none, (2) a little, (3) some, (4) quite a lot, and (5) very much; (f) during the past 4 weeks, how much did your physical health or emotional problems limit your usual social activities with family or friends? The answers are as follows: (1) could not do social activities, (2) quite a lot, (3) somewhat, (4) very little, (5) not at all; (g) during the past 4 weeks, how much have you been bothered by emotional problems? The responses are as follows: (1) extremely, (2) quite a lot, (3) moderately, (4) slightly, and (5) not at all; (h) during the past 4 weeks, how much did personal or emotional problems keep you from doing your usual work, school, or other daily activities? The responses are  as follows: (1) could not do daily activities, (2) quite a lot, (3) somewhat, (4) very little, and (5) not at all. The scale ranges from 8 to 42 with higher score demonstrating higher health-related quality of life.

Based on previous studies, the psychometric properties of the scale such as Cronbach alpha are generally very high [39, 42, 43, 46, 50, 51]. For instance, Wirtz et al. [39] reported a Cronbach alpha value of 0.918 for all items under the SF-8 Health Survey scale. In this study, the Cronbach alpha for all the items was 0.932 showing strong internal consistency.

### Principal independent variable

The focal independent variable of this study is caregiver burden. The World Health Organization Impact of Caregiving Scale [World Health Organization cited in 24] was used to assess caregiver burden. The scale is made up of 10 items with a 5-point Likert scale ranging from: 1 = None, 2 = Mild, 3 = Moderate, 4 = Severe, 5 = Extreme. The 10 items scale sought to find out from informal caregivers who have provided care for at least a year [24], if caregiving results in the following: 1 = “difficulty getting enough sleep”, 2 = “problem getting enough food to eat”, 3 = “not enough energy for extra work”, 4 = “do not know the correct care to provide for health problems of care recipients”, 5 = “cannot take care of health, ailment/chronic condition”, 6 = “unable to pay for medication/treatment for ailment/chronic condition alone”, 7 = “cannot visit friends and relatives as much as before”, 8 = “cannot share feelings about caregiving responsibility with others”, 9 = “experienced financial problems due to loss of income”, 10 = “experienced stigma or problems as a result of the care recipient's illness or death”. Considering this, we built a composite score which ranged from 10 to 50 with higher score showing higher caregiver burden. A Cronbach alpha value of 0.881 was reported indicating strong internal consistency.

### Control variables

Socio-ecological (which is considered here as socio-economic), cultural, demographic, and healthcare factors were controlled for. Specifically, age (years) (0 = 18–24, 1 = 25–34, 2 = 35–44, 3 = 45–54, 4 = 55–64, 5 = 65 or above), gender (0 = male, 1 = female), ethnicity (0 = Akan, 1 = non-Akan), religion (0 = Christianity, 1 = Islam, 2 = African traditional religion, 3 = no religion), living arrangement (that is whether the participants are living with the care recipient or not) (0 = no, 1 = yes), health insurance enrollment (0 = no, 1 = yes), place of residence (0 = rural, 1 = urban), marital status (0 = never married, 1 = currently married, 2 = separated/widowed/divorced), monthly income level (GH¢) (0 = less than 1000 [US$99.50 as at the time of the field survey, September 2022], 1 = 1000–1999, 2 = 2000 or above), education level (0 = no formal education, 1 = primary education, 2 = junior high school education, 3 = senior high school education, 4 = tertiary education), employment status (0 = unemployed, 1 = employed) and healthcare (0 = none, 1 = once, 2 = 2 times, 3 = 3 times, 4 = 4 times 5 = 5 or more times) of caregivers. All the control variables were considered as categorical. When we performed multi-collinearity analysis of the independent and control variables, we had a variance inflation factor less than 1.5 which suggests no strong multi-collinearity.

### Analytical strategy

With the aid of the SPSS software version 28 (IBM, Armonk, NY), we employed descriptive (such as mean, standard deviation, percentages, and frequencies) and inferential analytical techniques (such as Generalized Linear Models) to analyze our data. The sample characteristics of the participants, caregiver burden and health-related quality of life were analyzed using frequencies, percentages, mean and standard deviations. Generalized Linear Models were employed to establish association between caregiver burden, other control variables (such as age, gender, ethnicity, religion, health insurance enrollment, place of residence, marital status, income level, education level, employment status and healthcare utilization) and health-related quality of life. We employed Generalized Linear Models because the dependent variable (that is health-related quality of life) was continuous. In all, five models were fitted. Model 1 determined the association between caregiver burden and health-related quality of life. Model 2 included variable in Model 1 plus socio-economic variables. Model 3 added cultural variables plus all variables in Model 2. Model 4 included demographic factors and all variables in Model 3. The final Model (Model 5) consisted of healthcare variable and all variables in Model 4. We reported beta values and standard errors with a significance level of 0.05 or less.

## Results

### Sample characteristics of the participants

Socio-economic, cultural, demographic, and healthcare characteristics of the participants are reported in Table 1. Our results showed that 28.6% of the participants had no level of education, 66.4% were employed, 79.6% were living with the care recipient, 76.8% earned a monthly income of less than GH¢1000 (US$99.50 as at the time of the field survey, September 2022), 76.6% had not enrolled in a health insurance scheme, 76.2% were of Akan ethnicity, 80.7% were Christians and 27.7% were aged between 25 and 34 years. Further, 72.9% of the participants were females, 56.7% resided in urban areas and 50.6% had not sought healthcare for their health problems in the last year before the survey (see Table 1).

**Table 1:** Socio-economic, cultural, demographic and healthcare characteristics of the participants (*N* = 1853)

Variables	Category/response	%
Education level of caregivers	No formal education	28.6
	Primary	8.2
	Junior high school	24.0
	Senior high school	24.0
	Tertiary	15.2
Employment status of caregivers	Unemployed	33.6
	Employed	66.4
Living arrangements with care recipients	No	20.4
	Yes	79.6
Income level (GH¢) (Monthly) of caregivers	Less than 1000	76.8
	1000–1999	16.1
	2000 or above	7.1
Health insurance enrollment of caregivers	No	23.4
	Yes	76.6
Ethnicity of caregivers	Akan	76.2
	Non-Akan	23.8
Religion of caregivers	Christianity	80.7
	Islam	15.9
	African Traditional Religion	0.7
	No religion	2.7
Age (years) of caregivers	18–24	14.3
	25–34	27.7
	35–44	23.7
	45–54	19.9
	55–64	9.3
	65 or above	5.1
Gender of caregivers	Male	27.1
	Female	72.9
Place of residence of caregivers	Rural	43.3
	Urban	56.7
Healthcare utilization of caregivers (in the last year before the survey)	None	50.6
	Once	13.4
	2 times	17.6
	3 times	7.7
	4 times	3.2
	5 or more times	7.4

### Descriptive analysis of caregiver burden among informal caregivers of older adults

We report a descriptive analysis of prevalence of caregiver burden among informal caregivers of older adults in Table 2. Our analysis showed that 47.6% of the participants had no difficulty of getting enough sleep, 55.9% had no problem of getting enough food to eat, 49.8% had enough energy for extra work, 52.8% knew the correct care to provide for health problems of care recipients, 43.8% could take care of the health, ailment/chronic conditions of the care recipient and 30% were able to pay for medication/treatment for ailment/chronic condition alone. Also, 43.8% could visit friends and relatives as much as before, 49.9% were able to share their feelings about caregiving responsibility with others, 30.1% did not experience financial problems due to loss of income and 84.2% did not experience stigma or problems because of the care recipient’s illness. The mean values suggest that informal caregivers of older adults in this study experienced low to moderate caregiver burden (see Table 2).

**Table 2:** Prevalence of caregiver burden among informal caregivers of older adults  (*N* = 1853)

Variable/item	Response	Total %	Mean	SD
Difficulty getting enough sleep	None	47.6		
	Mild	16.1	2.13	1.285
	Moderate	17.8		
	Severe	13.2		
	Extreme	5.4		
Problem getting enough food to eat	None	55.9		
	Mild	13.0	2.13	1.348
	Moderate	13.5		
	Severe	10.8		
	Extreme	6.7		
Not enough energy for extra work	None	49.8	2.12	1.380
	Mild	15.2		
	Moderate	15.5		
	Severe	11.4		
	Extreme	8.0		
Do not know the correct care to provide for health problems of care recipients	None	52.8		
	Mild	12.3	2.55	1.608
	Moderate	13.1		
	Severe	14.1		
	Extreme	7.7		
Cannot take care of health, ailment/chronic condition	None	43.8		
	Mild	10.7	2.55	1.608
	Moderate	12.6		
	Severe	12.8		
	Extreme	20.1		
Unable to pay for medication/treatment for ailment/chronic condition alone	None	30.0		
	Mild	13.8		
	Moderate	18.9	2.84	1.520
	Severe	16.5		
	Extreme	20.8		
Cannot visit friends and relatives as much as before	None	43.8		
	Mild	13.4		
	Moderate	12.8	2.48	1.576
	Severe	11.2		
	Extreme	18.9		
Cannot share feelings about caregiving responsibility with others	None	49.9		
	Mild	11.7	2.34	1.573
	Moderate	10.2		
	Severe	11.1		
	Extreme	17.2		
Experienced financial problems due to loss of income	None	30.1		
	Mild	12.8	2.78	1.441
	Moderate	20.3		
	Severe	22.5		
	Extreme	14.2		
Experienced stigma or problems as a result of the care recipient's illness or death	None	84.2		
	Mild	6.7		
	Moderate	3.6	1.33	.886
	Severe	2.8		
	Extreme	2.7		
Reliability test items (Cronbach’s alpha based on standardized items)		10 (.881)		

### Descriptive analysis of health-related quality of life among informal caregivers of older adults

Descriptive analysis of health-related quality of life among informal caregivers of older adults is reported in Table 3. The results showed that 47.9% of the participants self-rated their health as very good, 50.1% indicated that physical health problems did not limit their usual physical activities, 52.1% reported that they did not have any difficulty because of physical health problems in doing their daily work and 44.5% did not experience bodily pains in the last 4 weeks. Also, 36% said they have “very much “energy in the past 4 weeks, 45.6% indicated that physical health or emotional problems did not limit their social activities with family or friends and 45.2% said that they were not bothered by emotional problems. Close to half  (46.8%) of the participants indicated that their personal or emotional problems did not keep them from doing their usual daily activities. In sum, the mean values of all the items demonstrate that the participants reported a relatively high health-related quality of life (see Table 3).

**Table 3:** Descriptive analysis of health-related quality of life among informal caregivers of older adults  (*N* = 1853)

Item	Response	%	Mean	SD
Overall, how would you rate your health during the past 4 weeks?	Very poor	0.2	4.9611	.87908
	Poor	1.3		
	Fair	3.9		
	Good	19.1		
	Very good	47.9		
	Excellent	27.7		
During the past 4 weeks, how much did physical health problems limit your usual physical activities (such as walking or climbing stairs)?	Could not do physical activities	0.3	4.3211	.82677
	Quite a lot	4.4		
	Somewhat	8.4		
	Very little	36.8		
	Not at all	50.1		
During the past 4 weeks, how much difficulty did you have in doing your daily work, both at home and away from home because of your physical health?	Could not do daily work	0.3	4.3260	.85414
	Quite a lot	5.0		
	Somewhat	8.5		
	A little bit	34.1		
	Not at all	52.1		
How much bodily pain have you had during the past 4 weeks?	Very severe	2.4	4.9498	1.29593
	Severe	4.8		
	Moderate	8.0		
	Mild	9.3		
	Very mild	30.9		
	None	44.5		
During the past 4 weeks, how much energy did you have?	None	1.0	4.1311	.85287
	Little	4.8		
	Some	10.4		
	Quite a lot	47.8		
	Very much	36.0		
During the past 4 weeks, how much did your physical health or emotional problems limit your usual social activities with family or friends?	Could not do social activities	0.3	4.2536	.84065
	Quite a lot	4.8		
	Somewhat	9.8		
	Very little	39.5		
	Not at all	45.6		
During the past 4 weeks, how much have you been bothered by emotional problems (such as feeling anxious, depressed, or irritable)?	Extremely	0.8	4.2029	.89687
	Quite a lot	4.9		
	Moderately	12.8		
	Slightly	36.3		
	Not at all	45.2		
During the past 4 weeks, how much did personal or emotional problems keep you from doing your usual work, school, or other daily activities?	Could not do daily activities	0.3	4.2779	.82610
	Quite a lot	4.3		
	Somewhat	9.7		
	Very little	39.0		
	Not at all	46.8		
Reliability test items (Cronbach’s alpha based on standardized items) [items (Cronbach's Alpha)]		[8 (.932)]		

### Regression analysis

Table 4 summarizes the main findings of the Generalized Linear Models for the relationship between caregiver burden and health-related quality of life among informal caregivers of older adults. Findings from Model 1 demonstrated a statistically significant association between caregiver burden and health-related quality of life. For instance, the study specifically revealed that caregiver burden was negatively associated with health-related quality of life (*β* = − 0.262, Standard Error [SE] = 0.3138, *p* value = 0.001). Suggesting that higher levels of caregiver burden were associated with decreased health-related quality of life and vice versa.  In Model 2, after adjusting for socio-economic status, the association between caregiver burden and health-related quality of life was still present. For instance, the study revealed that caregiver burden was negatively associated with health-related quality of life (*β* = − 0.271, SE = 0.0129, *p* value = 0.001). In Model 3, when cultural factors such as ethnicity and religion were added to all variables in Model 2, we found that caregiver burden was negatively associated with health-related quality of life (*β* = − 0.268, SE = 0.0129, *p* value = 0.001). In Model 4, when demographic factors were added to all variables in Model 3, we observed that caregiver burden negatively predicted health-related quality of life  (*β *=  − 0.262, SE = 0.0127, *p* value = 0.001). In the final model, when we added healthcare factors to all variables in Model 4, we observed that caregiver burden was negatively associated with health-related quality of life (*β* = − 0.286, SE = 0.0123, *p* value = 0.001).

**Table 4:** Caregiver burden and health-related quality of life among informal caregivers of older adults

Variables	Model 1 *β* (SE)	Model 2 *β* (SE)	Model 3 *β* (SE)	Model 4	Final model	Collinearity statistics	
						Tolerance	VIF
Socio-ecological factors							
Caregiver burden	− .262 (.3138)***	− .271 (.0129)***	− .268 (.0129)***	− .262 (.0127)***	− .286 (.0123)***	.886	1.129
Socio-economic status of caregivers							
Education							
No formal education		− 2.939 (.3937)***	− 2.608(.3989)***	− 1.429 (.4251)***	− 1.204 (.4085)**	.722	1.385
Primary		− 3.428 (.5296)***	− 3.201 (.5291)***	− 2.673 (.5306)***	− 2.390 (.5099)***		
Junior high school		− 1.831 (.4031)***	− 1.754(.4014)***	− 1.544 (.4045)***	− 1.113 (.3903)**		
Senior high school		− .345(.4023)	− .292 (.4001)	− .581 (.3960)	− .451 (.3804)		
Tertiary (ref)		0.00	0.00	0.00	0.00		
Employment							
Unemployed		404 (.2647)	.392 (.2632)	− .192 (.2798)	-.373 (.2694)	.903	1.108
Employed (ref)		0.00	0.00	0.00	0.00		
Living arrangements							
No		.005 (.3064)	.023(.3048)	.127 (.2990)	.226 (.2876)	.965	1.036
Yes (ref)		0.00	0.00	0.00	0.00		
Income (GH¢) (monthly)							
Less than 1000		.214(.4843)	.320 (.4819)	− .090 (.4720)	.049 (.4534)	.936	1.068
1000–1999		.002 (.5500)	.011 (.5471)	.015 (.5339)	− .042 (.5129)		
2000 or above (ref)		0.00	0.00	0.00	0.00		
Enrolment in health insurance scheme							
No		.365 (.2984)	.410 (.2975)	.076 (.2967)	− .339 (.2872)	.878	1.139
Yes (ref)		0.00	0.00	0.00	0.00		
Cultural factors							
Ethnicity							
Akan (ref)			.775 (.4014)	1.117 (.3935)**	.802 (.3789)*	.798	1.253
Non-Akan			0.00	0.00	0.00		
Religion							
Christianity			2.775 (.7584)***	3.082 (.7464)***	2.572 (.7234)***	.775	1.290
Islam			2.599 (.8460)**	2.798 (.8273)***	2.244 (.8011)**		
African Traditional Religion			.488 (1.6295)	.738 (1.5897)	1.626 (.8011)		
No religion (ref)			0.00	0.00	0.00		
Demographic factors of caregivers							
Age (years)							
18–24				4.371 (.6456)***	2.960 (.6306)***	.763	1.311
25–34				3.023 (.5931)***	1.728 (.5794)**		
35–44				2.615 (.5940)***	1.604 (.5764)**		
45–54				.979(.5997)	.198 (.5796)		
55–64				.213 (.6561)	− .364 (.6322)		
65 or above (ref)				0.00	0.00		
Gender							
Male				.468 (.2826)	.246 (.2721)	.888	1.126
Female (ref)				0.00	0.00		
Place of residence							
Rural				.096 (.2470)	.102 (.2377)	.927	1.079
Urban (ref)				0.00	0.00		
Healthcare factors of caregivers							
Healthcare utilization							
None					4.786 (.4610)***		
Once					2.985 (.5275)***		
2 times					2.502 (.4989)***	.880	1.136
3 times					1.599 (.5832)**		
4 times					1.635 (.7556)*		
5 or more times (ref)					0.00		
Model fitness							
Likelihood ratio Chi-square (*p* value)	383.983***	490.024***	514.213***	614.767***	766.808***		
Wald Chi-square (*p* value)	17,377.111***	10,447.535***	4983.153***	4882.900***	5190.915***		

*Test is significant at the 0.05 level

**Test is significant at the 0.01 level

***Test is significant at the 0.001 level

 Other socio-economic, cultural, demographic, and healthcare factors were associated with health-related quality of life. Having no formal education (*β* = − 1.204, SE = 0.4085, *p* value = 0.01), primary level of education (*β* = − 2.390, SE = 0.5099, *p* value = 0.001) or junior high school education (*β* = − 1.113, SE = 0.3903, p = 0.01) was strongly associated with decreased health-related quality of life compared to those with tertiary level of education. Also, being of Akan ethnicity was associated with increased health-related quality of life (*β* = 0.802, SE = 0.3789, *p* value = 0.05). Being affiliated with Christianity (*β* = 2.572, SE = 0.7234, *p* value = 0.001) or Islam (*β* = 2.244, SE = 0.8011, *p* value = 0.01) religion was associated with increased health-related quality of life. More importantly, being between the ages of 18–24 (*β* = 2.960, SE = 0.6306, *p* value = 0.001), 25–34 (*β* = 1.728, SE = 0.5794, *p* value = 0.01) or 35–44 (*β* = 1.604, SE = .5764, *p* value = 0.01) years was associated with increased health-related quality of life compared to those who were 65 years or above. Last, we found that not using healthcare services in the past year before the survey was strongly associated with increased health-related quality of life compared to those who utilized healthcare services five or more times in the past year (*β* = 4.786, SE = . 4610, *p* value = 0.001).

### Discussion and implications

The findings suggest that the health-related quality of life among informal caregivers of older people in the Ashanti Region of Ghana is significant and should be considered as part of efforts to strengthen caregiving for older adults. This has significant practical and policy implications given that caregiving within the informal domain is prevalent in SSA [21] due to strong cultural norms that sanction caregiving for older people [23]. Our findings raise significant awareness of the health-related quality of life among informal caregivers who provide care to older people in Ghana.

We predicted that caregiver burden would be negatively associated with health-related quality of life. This hypothesis was robust even after accounting for key socio-economic, cultural, demographic and healthcare factors. In general, the findings corroborate existing evidence in Europe [32], and Asia [33]. For example, Hsiao and colleagues [33] report that primary caregivers of older adults who experienced higher levels of caregiver burden also reported poor health-related quality of life. Aside from corroborating global evidence, the findings have some implications which are unique to the Ghanaian context. First, considering that caregiving for older adults is common (anecdotal evidence suggests that 1 in 3 Ghanaian informal caregivers provide care to older people) and culturally sanctioned in Ghana, evidence of a robust negative relationship between caregiver burden and health-related quality of life suggests that the majority of Ghanaians informal caregivers of older adults would have poor health-related quality of life. Second, evidence on the psychological [24] and emotional challenges experienced by informal caregivers in Ghana [23] suggests that most caregivers lack knowledge about self-care and other proactive measures that might improve their health-related quality of life. Theoretically, it is highly expected that a caregiver, who is a close relative of an older person will experience some levels of emotional discomfort leading to severe caregiver burden due to the emotional bond and connection between them. Hence, caregiver’s failure to engage in self-care practice and socialization activities (as reported in Agyemang-Duah et al. [19]) could increase their caregiver burden, trigger trauma, and affect their health-related quality of life. As a result, it is highly recommended that the health-related quality of life among informal caregivers of older people is given special consideration in both policy and practice.

Programmes that increase caregivers’ knowledge about self-care, broaden their social connection and participation in community social activities should be created and strengthened to ensure that caregivers with severe caregiver burden have some breathing space and avenues to recharge. Healthcare policies should provide opportunities for informal caregivers of older people to be included in periodic check-ups to ensure that both informal caregivers and their carers are in good medical condition. Our findings showed that caregivers rate of healthcare use is significantly associated with health-related quality of life. Suggesting that interventions that will boost healthcare utilization among informal caregivers could be protective against poor health-related quality of life. We are more likely to create a vicious cycle of caregiver burden, whereby anyone who assumes primary responsibilities as an informal caregiver ends-up as a carer who requires care, if the caregiver burden and the associated poor health-related quality of life among informal caregivers of older adults are not addressed. However, we can make significant changes in the lives of older people and their caregivers if the health-related quality of life among informal caregivers of older adults is given much attention both in policy and practice.

Following previous literature [28, 31, 35], we found that caregivers levels of education, ethnicity and age significantly predicted health-related quality of life. Specifically, we found that lower levels of education were associated with decreased health-related quality of life. While this may be intuitive, since compared to caregivers with higher levels of education, those with low educational attainments are less likely to practice self-care, utilize healthcare, and less likely to be aware of the symptoms of caregiver burden and the associated sequelae. Empirically, the findings confirm that educational awareness can play a significant role in efforts to ameliorate the impact of caregiver burden on the health-related quality-of-life among informal caregivers of older adults. Indeed, education on the symptoms of severe caregiver burden, self-care practice, and promotion of healthcare utilization would help to improve caregiver’s knowledge and spur them to engage in proactive measures, such as medical check-ups. Gelée and Andualem [28] also found that health-related literacy is associated with quality of life among family caregivers, although their study only focused on caregivers of people with mental illness in Ethiopia.

Findings on the relationship among ethnicity, religion, and health-related quality of life among informal caregivers of older people highlight the importance of social support and social connections in the lives of informal caregivers. Religion and ethnicity create avenues for people to establish social and spiritual connections which are significant for sourcing social support in times of difficulty. For example, a caregiver with severe physical burden may benefit from caregiving support provided by close members within their religious and social groups. Previous studies in Ethiopia [28] and Ghana [31] document social support and relationships among the crucial measures required to prevent the negative impacts of caregiver burden. On the positive side, the evidence confirms that in addition to medical interventions, strengthening social and religious connections can be useful in addressing the negative effects of severe caregiver burden on health-related quality of life among informal caregivers of older adults.

### Strengths and limitations of the study

This study contributes to empirical, methodological, and theoretical knowledge. This is the first Ghanaian study to examine the relationship between caregiver burden and health-related quality of life among informal caregivers of older adults. The specific empirical contribution of this study is that caregiver burden is negatively associated with health-related quality of life. Beyond caregiver burden, another empirical contribution of this study is that some socio-economic, (such as education), cultural (such as ethnicity and religion), demographic (such as age) and healthcare (such as healthcare utilization) factors are significantly associated with health-related quality of life. Theoretically, we are the first to employ the conceptual model of health-related quality of life to understand the relationship between caregiver burden and health-related quality of life among informal caregivers of older adults in Ghana. We thus confirm that our findings to some extent support the conceptual model of health-related quality of life in the Ghanaian context. Third, the methodological contributions/strengths of this study are premised on the following: (1) the use of a large sample size of 1853 informal caregivers from 39 communities made up of 18 rural and 21 urban communities in 13 districts in Ghana; (2) the use of standardized and validated instruments with high Cronbach alpha values to measure caregiver burden and health-related quality of life ensured that our results are internally consistent/reliable; (3) the division of the study area into diverse geographical zones of south, middle and north made our results geographically representative of the Ashanti Region. Despite these strengths/contributions of our study, the following limitations are also acknowledged. First, this study used a cross-sectional survey design which limits our ability to draw causal inferences between caregiver burden and health-related quality of life. Second, we recruited our participants from one region which might limit the generalization and representativeness of our findings to other regions of Ghana. Third, we used snowball sampling to recruit our participants which has the potential to restrict randomization. Last, our data were self-reported, so minor recall bias is possible. Taken together the above strengths/limitations, we suggest that future studies should employ a longitudinal design from more than one region in Ghana or more than one country in SSA to investigate the relationship between caregiver burden and health-related quality of life among informal caregivers of older adults. We also believe that in-depth qualitative studies are needed to understand the various emotional tensions that motivate informal caregivers to provide care for older adults in developing countries including Ghana. 

## Conclusion

In this study, we examined the relationship between caregiver burden and health-related quality of life among informal caregivers of older adults. Consistent with our hypothesis, we found that caregiver burden was negatively associated with health-related quality of life. We argue that aside from caregiver burden, socio-economic, demographic, cultural and healthcare factors were associated with health-related quality of life. Our findings partially support the conceptual model of health-related of quality which highlights that socio-ecological (including caregiver burden and socio-economic), demographic, cultural and healthcare factors explain health-related quality of life. These findings are important for health actors including practitioners to understand factors associated with health-related quality of life among informal caregivers of older adults. Guided by our findings, we suggest that health and social policy measures to improve health-related quality of life among informal caregivers of older adults need to consider caregiver burden as well as other significant socio-ecological, cultural, demographic, and healthcare factors. We further recommend that since this study was purely quantitative, future research should use mixed and longitudinal methods to capture both quantitative and qualitative factors associated with health-related quality of life among informal caregivers of older adults. Such investigations would be useful to inform robust and comprehensive policy development to improve health-related quality of life among informal caregivers of older adults in Ghana and other developing countries.

## Data Availability

The datasets used and/or analyzed during the current study are available from the corresponding author on reasonable request.

## References

[1] United Nations. Population facts. United Nations, Department of Economic and Social Affairs Population Division, No. 2016/1;2016.

[2] Ghana Statistical Service. The elderly in Ghana. 2010 population & housing census report. Author, Accra, Ghana;2013.

[3] Agyemang-Duah W, Asante D, Oduro Appiah J, Morgan KA, Mensah IV, Peprah P, Mensah AA (2023). System, institutional, and client-level factors associated with formal healthcare utilisation among older adults with low income under a social protection scheme in Ghana. Arch Public Health.

[4] Audain K, Carr M, Dikmen D, Zotor F, Ellahi B (2017). Exploring the health status of older persons in Sub-Saharan Africa. Proc Nutr Soc.

[5] Gyasi RM, Phillips DR (2020). Aging and the rising burden of noncommunicable diseases in sub-Saharan Africa and other low-and middle-income countries: a call for holistic action. Gerontologist.

[6] Ayernor PK (2012). Diseases of ageing in Ghana. Ghana Med J.

[7] Agyemang-Duah W, Peprah C, Arthur-Holmes F (2019). Prevalence and patterns of health care use among poor older people under the Livelihood Empowerment Against Poverty program in the Atwima Nwabiagya District of Ghana. Gerontol Geriatr Med.

[8] Minicuci N, Biritwum RB, Mensah G, Yawson AE, Naidoo N, Chatterji S, Kowal P (2014). Sociodemographic and socioeconomic patterns of chronic non-communicable disease among the older adult population in Ghana. Glob Health Action.

[9] Awoke MA, Negin J, Moller J, Farell P, Yawson AE, Biritwum RB, Kowal P (2017). Predictors of public and private healthcare utilization and associated health system responsiveness among older adults in Ghana. Glob Health Action.

[10] Banerjee C (2015). Multi-morbidity-older adults need health care that can count past one. Lancet.

[11] Adatara P, Amooba PA (2021). A qualitative exploration of barriers to the utilisation of outpatient healthcare services among older persons in the Ho Municipality of Volta Region of Ghana. Int J Afr Nurs Sci.

[12] Agyemang-Duah W, Peprah C, Peprah P (2019). Barriers to formal healthcare utilisation among poor older people under the livelihood empowerment against poverty programme in the Atwima Nwabiagya District of Ghana. BMC Public Health.

[13] World Health Organization (WHO). Integrated care for older people (ICOPE). Guidelines on community-level interventions to manage declines in intrinsic capacity. World Health Organization, Geneva;2017. 29608259

[14] Aboderin IA, Beard JR (2015). Older people's health in sub-Saharan Africa. The Lancet.

[15] Keating N (2011). Critical reflections on families of older adults. Adv Gerontol.

[16] Roth DL, Fredman L, Haley WE (2015). Informal caregiving and its impact on health: a reappraisal from population-based studies. Gerontologist.

[17] Biliunaite I, Kazlauskas E, Sanderman R, Andersson G (2022). Informal caregiver support needs and burden: a survey in Lithuania. BMJ Open.

[18] Law S, Ormel I, Babinski S, Kuluski K, Quesnel-Vallée A (2021). “Caregiving is like on the job training but nobody has the manual”: Canadian caregivers’ perceptions of their roles within the healthcare system. BMC Geriatr.

[19] Agyemang-Duah W, Mensah CM, Peprah P, Arthur F, Addai B, Abalo EM (2019). Informal health care: examining the role of women and challenges faced as caregivers in rural and urban settings in Ghana. J Public Health.

[20] World Health Organization. A glossary of terms for community health care and services for older persons. WHO centre for health development ageing and health technical report, vol 5;2004.

[21] Addo R, Agyemang SA, Tozan Y, Nonvignon J (2018). Economic burden of caregiving for persons with severe mental illness in sub-Saharan Africa: A systematic review. PLoS ONE.

[22] Akosile CO, Banjo TO, Okoye EC, Ibikunle PO, Odole AC (2018). Informal caregiving burden and perceived social support in an acute stroke care facility. Health Qual Life Outcomes.

[23] Okai R. Burden of caregiving on informal caregivers of stroke survivors in the Western Region. Master’s dissertation, University of Ghana, Accra, Ghana;2019.

[24] Sanuade OA, Boatemaa S (2015). Caregiver profiles and determinants of caregiving burden in Ghana. Public Health.

[25] Kim A, Woo K (2022). Gender differences in the relationship between informal caregiving and subjective health: the mediating role of health promoting behaviors. BMC Public Health.

[26] Chan CY, De Roza JG, Ding GTY, Koh HL, Lee ES (2023). Psychosocial factors and caregiver burden among primary family caregivers of frail older adults with multimorbidity. BMC Prim Care.

[27] Yuen EY, Wilson CJ (2021). The relationship between cancer caregiver burden and psychological outcomes: the moderating role of social connectedness. Curr Oncol.

[28] Gelée H, Andualem A (2022). Quality of life and associated factors among family caregivers of individuals with psychiatric illness at DRH, South Wollo, Ethiopia, 2020. Sci Rep.

[29] Opoku-Boateng YN, Kretchy IA, Aryeetey GC, Dwomoh D, Decker S, Agyemang SA (2017). Economic cost and quality of life of family caregivers of schizophrenic patients attending psychiatric hospitals in Ghana. BMC Health Serv Res.

[30] Dawson CP, Aryeetey GC, Agyemang SA, Mensah K, Addo R, Nonvignon J (2020). Costs, burden and quality of life associated with informal caregiving for children with Lymphoma attending a tertiary hospital in Ghana. Int J Care Coord.

[31] Boakye H, Nsiah A, Bello AI, Quartey JN (2017). Burden of care and quality of life among caregivers of stroke survivors: Influence of clinical and demographic variables. Br J Med Med Res.

[32] Valcárcel-Nazco C, Ramallo-Fariña Y, Linertová R, Ramos-Goñi JM, García-Pérez L, Serrano-Aguilar P (2022). Health-related quality of life and perceived burden of informal caregivers of patients with rare diseases in selected European countries. Int J Environ Res Public Health.

[33] Hsiao CY, Lu HL, Tsai YF (2020). Caregiver burden and health-related quality of life among primary family caregivers of individuals with schizophrenia: a cross-sectional study. Qual Life Res.

[34] Schandl A, Ringborg C, Mälberg K, Johar A, Lagergren P (2022). Caregiver burden and health-related quality of life among family caregivers of oesophageal cancer patients: a prospective nationwide cohort study. Acta Oncol.

[35] Sandstedt P, Littorin S, Cröde Widsell G, Johansson S, Gottberg K, Ytterberg C (2018). Caregiver experience, health-related quality of life and life satisfaction among informal caregivers to patients with amyotrophic lateral sclerosis: a cross-sectional study. J Clin Nurs.

[36] Faronbi JO, Olaogun AA (2017). The influence of caregivers’ burden on the quality of life for caregivers of older adults with chronic illness in Nigeria. Int Psychogeriatr.

[37] Ashing-Giwa KT (2005). The contextual model of HRQoL: a paradigm for expanding the HRQoL framework. Qual Life Res.

[38] Agyemang-Duah W, Rosenberg MW. Healthcare utilization among informal caregivers of older adults in the Ashanti region of Ghana: a study based on the health belief model. Archives of Public Health. 2023; 81(1):1-18 10.1186/s13690-023-01200-5 PMC10591341 37872631

[39] Wirtz MA, Schulz A, Brähler E (2021). Confirmatory and bi-factor analysis of the Short Form Health Survey 8 (SF-8) scale structure in a German general population sample. Health Qual Life Outcomes.

[40] Ware JE, Snow KK, Kosinski M, Gandek B. SF-36 health survey. Manual and interpretation guide, 2;1993.

[41] Ware JE, Kosinski M, Dewey JE, Gandek B (2001). How to score and interpret single-item health status measures: a manual for users of the SF-8 health survey. Lincoln, RI QualityMetric Incorporated.

[42] Beierlein V, Morfeld M, Bergelt C, Bullinger M, Brähler E (2012). Messung der gesundheitsbezogenen Lebensqualität mit dem SF-8. Diagnostica.

[43] Namjoo S, Mirzaei M, Foroughan M, Harouni GG (2021). Psychometric properties of the Short Form-8 Health Survey (SF-8) among diabetes and non-diabetes Iranian older people. Health Promot Perspect.

[44] Yiengprugsawan VS, Kelly M, Tawatsupa B, Maggino F (2021). SF-8™ Health Survey. Encyclopedia of quality of life and well-being research.

[45] Onagbiye SO, Moss SJ, Cameron M (2018). Validity and reliability of the Setswana translation of the Short Form-8 health-related quality of life health survey in adults. Health SA Gesondheid.

[46] Lang L, Zhang L, Zhang P, Li Q, Bian J, Guo Y (2018). Evaluating the reliability and validity of SF-8 with a large representative sample of urban Chinese. Health Qual Life Outcomes.

[47] Ellert U, Lampert T, Ravens-Sieberer U (2005). Measuring health-related quality of life with the SF-8: normal sample of the German population. Bundesgesundheitsblatt-Gesundheitsforschung-Gesundheitsschutz.

[48] Wang P, Fu AZ, Wee HL, Lee J, Tai ES, Thumboo J, Luo N (2013). Predicting preference-based SF-6D index scores from the SF-8 health survey. Qual Life Res.

[49] Lefante JJ, Harmon GN, Ashby KM, Barnard D, Webber LS (2005). Use of the SF-8 to assess health-related quality of life for a chronically ill, low-income population participating in the Central Louisiana Medication Access Program (CMAP). Qual Life Res.

[50] Tomás JM, Galiana L, Fernández I (2018). The SF–8 Spanish version for health-related quality of life assessment: psychometric study with IRT and CFA models. Span J Psychol.

[51] Roberts B, Browne J, Ocaka KF, Oyok T, Sondorp E (2008). The reliability and validity of the SF-8 with a conflict-affected population in northern Uganda. Health Qual Life Outcomes.

